# Efficacy and safety of ivermectin in the treatment of mild to moderate COVID-19 infection: a randomized, double-blind, placebo-controlled trial

**DOI:** 10.1186/s13063-022-06649-3

**Published:** 2022-08-26

**Authors:** Anan Manomaipiboon, Kittisak Pholtawornkulchai, Sujaree Poopipatpab, Swangjit Suraamornkul, Jakravoot Maneerit, Wiroj Ruksakul, Uraporn Phumisantiphong, Thananda Trakarnvanich

**Affiliations:** Faculty of Medicine, Vajira Hospital, Navamindradhiraj University, Dusit, Bangkok, Thailand

**Keywords:** Efficacy, Ivermectin, COVID-19, Randomized controlled trial, SARS-CoV-2, RT-PCR

## Abstract

**Background:**

The emergent outbreak of coronavirus disease (COVID-19), caused by severe acute respiratory syndrome coronavirus 2 (SARS-CoV-2), has emphasized the requirement for therapeutic opportunities to overcome this pandemic. Ivermectin is an antiparasitic drug that has shown effectiveness against various agents, including SARS-CoV-2. This study aimed to assess the efficacy of ivermectin treatment compared with the standard of care (SOC) among people with mild to moderate COVID-19 symptoms.

**Methods:**

In this randomized, double-blind, placebo-controlled, single-center, parallel-arm, superiority trial among adult hospitalized patients with mild to moderate COVID-19, 72 patients (mean age 48.57 ± 14.80 years) were randomly assigned to either the ivermectin (*n*=36) or placebo (*n*=36) group, along with receiving standard care. We aimed to compare the negativity of reverse transcription polymerase chain reaction (RT-PCR) result at days 7 and 14 of enrolment as the primary outcome. The secondary outcomes were duration of hospitalization, frequency of clinical worsening, survival on day 28, and adverse events.

**Results:**

At days 7 and 14, no differences were observed in the proportion of PCR-positive patients (RR 0.97 at day 7 (*p*=0.759) and 0.95 at day 14 (*p*=0.813). No significant differences were found between the groups for any of the secondary endpoints, and no adverse events were reported.

**Conclusion:**

No difference was found in the proportion of PCR-positive cases after treatment with ivermectin compared with standard care among patients with mild to moderate COVID-19 symptoms. However, early symptomatic recovery was observed without side effects.

**Trial registration:**

ClinicalTrials.gov NCT05076253. Registered on 8 October 2021, prospectively.

**Supplementary Information:**

The online version contains supplementary material available at 10.1186/s13063-022-06649-3.

## Introduction

The newly emerged coronavirus disease (COVID-19) has spread globally, with recent estimates of more than 236 million cases and 4.8 million deaths reported as of November 2021 [1]. Therapeutic approaches are required to improve outcomes among patients with COVID-19 because no antiviral agent has yet been proved to be conclusively beneficial in treating COVID-19, especially among patients presenting mild to moderate severity. Despite the urgent need to find an effective antiviral treatment for COVID-19 through randomized controlled studies, certain agents are being used globally based on either in vitro or observational studies. The most frequently used agents in Thailand and globally include andrographolide, hydroxychloroquine, lopinavir/ritonavir, favipiravir, and remdesivir. Ultimately, none have proved to be efficacious or safe.

Interest has been growing regarding the antiparasitic drug, ivermectin, which was previously studied for its antiviral, antiinflammatory, and anticancer actions [2]. Ivermectin was also reported to have an in vitro activity against severe acute respiratory syndrome 2 (SARS-CoV-2), the virus that causes COVID-19 [3]. Its antiviral properties include its action on importin 2/β1-mediated nuclear transport. Ivermectin prevents the binding of viral proteins to importin 2/β1, rendering the viral proteins unable to enter the nucleus and subsequently cause infection [4]. It acts at different viral protein binding sites, thereby reducing viral replication. The blockage of the transport of viral proteins from the cytosol to the nucleus may be one mechanism of action.

Several clinical studies have found a beneficial effect of ivermectin in treating COVID-19 [5–9]. One recent meta-analysis found that ivermectin reduced the risk of death compared with no ivermectin (average risk ratio 0.38, 95% confidence interval 0.19–0.73; *n* = 2438; *I*^2^ = 49%; moderate-certainty evidence) [10]. However, some studies did not find a significant difference between the group receiving ivermectin and the control group [11] including the systematic review from the Cochrane COVID-19 Study Register, Web of Science (Emerging Citation Index and Science Citation Index), and medRxiv [12]. Popp et al. were uncertain about the efficacy and safety of ivermectin used to treat or prevent COVID-19. To date, controlled trials evaluating ivermectin for treating COVID-19 are scarce. Because ivermectin is reportedly safe, with side effects of less than 1%, it would be essential to conduct a clinical trial with ivermectin for treating patients with COVID-19. This study aimed to establish the efficacy of ivermectin to treat patients with COVID-19 presenting mild to moderate symptoms, compared with usual care alone.

## Methods

### Study population

The study population included 72 patients with COVID-19, confirmed using a positive RT-PCR, with mild to moderate symptoms, within 72 h of a positive result or onset of symptoms. This study was approved by the Vajira Institutional Review Board no. 171/64 and was conducted in compliance with the Declaration of Helsinki and Good Clinical Practice Guidelines. Written informed consent was obtained from all patients. More details of the trial can be found in the protocol (Supplement 1). The inclusion criteria comprised adult men and women aged 18 to 80 years, nonpregnant or breast-feeding women, and mild to moderate symptoms as defined by the World Health Organization (WHO) severity score for COVID-19 [13]. Mild disease was defined as cough, runny nose, anosmia, fever, and diarrhea without dyspnea or tachypnea, and moderate disease was defined as pneumonia with oxygen saturation >90%. All patients were admitted to the hospital.

The patients were excluded if they were allergic to ivermectin, had the potential for a drug-to-drug interaction with ivermectin, such as tamoxifen or warfarin; were previously treated with ivermectin in the last 7 days; had received any herbal medicine; had severe chronic illness (severe congestive heart failure, chronic kidney disease stages 4 to 5, chronic liver disease or had hepatic dysfunction or liver function test results more than 1.5 times the normal level, terminal cancer); had concurrent bacterial infection; or were unwilling to participate in the trial. Patients with severe symptoms, likely due to cytokine release syndrome, uncontrolled comorbidities, and immunocompromised status were also excluded. No important changes were made to methods or trial outcomes after trial commencement. Also, no interim analyses or discontinued rules applied to the trial.

### Sample size calculation

The sample size calculation was based on a related reference study [14]. The number of 25 patients per group in the comparison of two proportions was calculated to have 90% power at a two-sided significance level of 0.05, allocation ratio 1:1 using continuity calculation. We used Stata, Version 16.0 to detect the proportion of patients with positive PCR at day 7 in the intervention and control groups of 9.8% (4/41; p1 = 0.098) and 55.6% (25/45; p2 = 0.556), respectively. The sample size was inflated to 36 participants per group (72 in total) to account for a possible 30% loss-to-follow-up, noncompliance, and drop-out.

### Study design and intervention

This study constituted a randomized, double-blind, placebo-controlled trial. This randomized, single-center, parallel-arm, superiority trial among adults was conducted at the Faculty of Medicine, Vajira Hospital, Navamindradhiraj University, from September 2021 to November 2021.

**Fig. 1:**
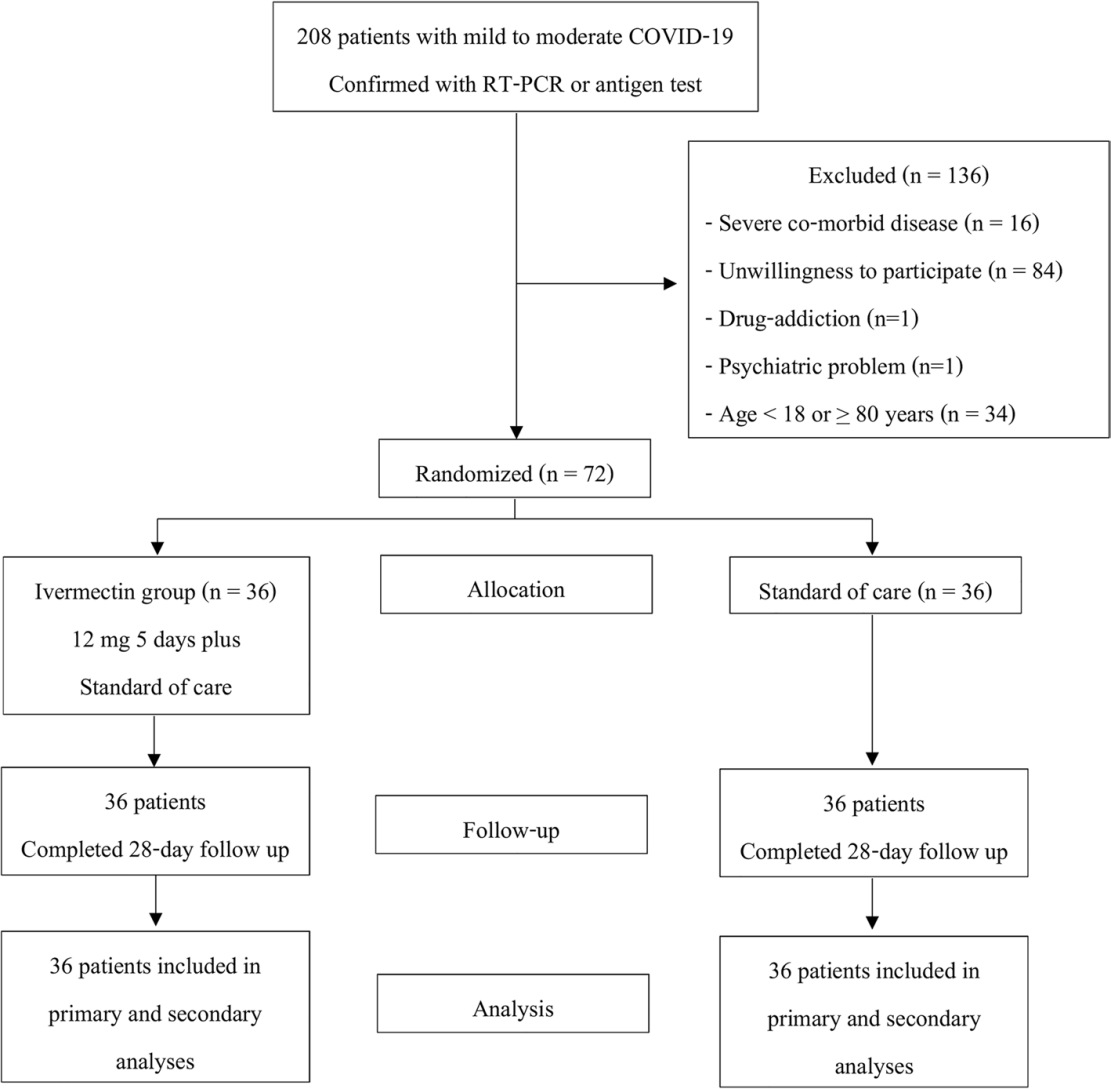
Study protocol and randomization

The patients were randomized in a permuted block of four in a randomized sequence prepared by a pharmacist, who was unblinded, in Microsoft Excel [15]. Allocation assignment was concealed from the investigators and patients. The patients were allocated in one of the two groups: group A (ivermectin arm) or group B (control arm), as shown in Fig. 1. The patients were randomized in a 1:1 ratio. Group A received 12 mg ivermectin daily for 5 days, as recommended by related studies [14], along with standard care. Group B received standard care alone, including favipiravir or andrographolide, corticosteroids, cetrizine, and paracetamol. No changes were made in the protocol after recruitment. Ivermectin was provided by the pharmacist by bottle. Patients were asked to take the investigational product on an empty stomach, except on the first study day, when administered after the postitive test result was confirmed.

### Clinical, laboratory, and virological monitoring

The study coordinator reviewed the patient’s history to screen for eligibility. The potential study participants were contacted by telephone to obtain informed consent. Eligible patients underwent physical examination by the doctor in the ward. Baseline characteristics, such as age, sex, comorbidities, duration of symptoms, and disease severity on admission, were recorded at the time of enrollment. All patients were confirmed as having COVID-19 using a baseline nasopharyngeal swab for RT-PCR. A follow-up RT-PCR was performed on days 7 and 14 following drug intervention to estimate the change in viral load. Complete blood count, renal and liver function tests, C-reactive protein, D dimer, and chest radiography were performed the day of enrollment and day 14. Patients were contacted via telephone by the research team every day through day 14. On day 28, a telephonic interview was performed for the final questions pertaining to general health, well-being, and the possible development of side effects after treating with ivermectin.

### Processing and analysis of respiratory samples

Nasopharyngeal swabs were collected from suspected COVID-19 cases by trained medical technologists. The swabs were stored in 2 mL of viral transport media (VTM) (Dewei Medical Equipment Co., Ltd., China), transported at 4 °C, and processed within 4 h at the Biomolecular Unit, central laboratory of Vajira Hospital. Viral RNA extraction was performed on each VTM sample using the commercial kit (Zybio Nucleic Acid Extraction Kit) on automated nucleic acid extraction system (magnetic bead method) (Zybio Inc., China), according to the manufacturer’s instructions. RT-PCR tests were run on a Slan 96P Real-Time PCR System using a 2019-nCoV Nucleic Acid Diagnostic Kit (Sansure Biotech Inc.). The kit is designed to detect N and ORF1 ab genes of SARS-CoV-2, along with one housekeeping gene as the internal amplification control. A 40 μL reaction contained 26 μl of reaction buffer, 4 μl of 2019-nCoV-PCR-Enzyme Mix, and 10 μl of RNA. Thermal cycling was performed at 50 °C for 30 min for reverse transcription and one cycle at 95 °C for 1 min. Then 45 cycles at 95 °C for 15 s and at 60 °C for 31 s were performed and analyzed using ABI 7500 Software. A positive RT-PCR result was defined when both target genes reached a cycle threshold (Ct) of <40.

### Outcome measurement

The primary outcome was to evaluate the efficacy of ivermectin in viral clearance of SARS-CoV-2 on days 7 and 14 after intervention, and compare that to placebo. The secondary outcomes were duration of hospitalization, frequency of clinical worsening, need for mechanical ventilation, all-cause mortality in both groups, survival on day 28, and adverse events in the study group.

### Statistical analysis

Data were analyzed according to the intention-to-treat principle. All descriptive data were expressed as mean (standard deviation) and frequency (percentage). Comparisons between the treatment group were determined using the Student *t* test for parametric continuous variables or the Mann–Whitney *U* for nonparametric continuous variables, as appropriate, and by the Pearson *X*^2^ test for categorial variables. Comparisons between the mean duration of viral clearance and duration of hospitalization were evaluated by the independent *t*-test or Mann–Whitney *U* test, as appropriate. Univariate analysis of the primary mortality outcome and comparisons between the treatment groups were determined using the chi-squared test. The primary end point of time from randomization to day 28 with ivermectin versus placebo was assessed using a Kaplan–Meier plot and compared with a long rank test. The hazard ratio and 95% confidence interval for the cumulative incidence of clinical worsening in both the treatment groups were estimated using the Cox proportional hazards model. Statistical significance was set as *P*<0.05, and all tests were two-tailed. Statistical analysews were performed using STATA, Version 18.1 (stata group).

## Results

### Baseline demographic and clinical characteristics

Between 10 October and 15 December 2021, 208 patients with mild to moderate COVID-19 symptoms within 3 days of symptoms onset were assessed for eligibility. Of the 208 assessed individuals, 134 were excluded due to severe comorbid diseases such as asthma and active malignancies, age-related ineligibility, and unwillingness to participate. Two patients withdrew their consent before the study due to drug addiction and psychiatric problems. The remaining target recruitment of 72 patients was equally randomized to either the ivermectin plus standard care (*n*=36) group or the placebo plus standard care (*n*=36) group (Fig. 1). The mean age of all the enrolled cases was 48.57± 14.80 years, and patients in both groups were balanced in demographic and disease characteristics at baseline (Table 1). The mean age of cases in the control and intervention arms did not significantly differ. The majority of patients in both the control and intervention groups were female. The main concomitant diseases were hypertension (49%), dyslipidemia (34%), and diabetes mellitus (23%). The biochemical parameters did not significantly differ between both groups and were all within normal limits (Table 1S, Supplemental File). The trial ended when the recruitment was achieved at the target or whenever the patients met criteria for withdrawal.

**Table 1:** General characteristics of the patients

Variables	Total (***n*** = 72)		Treatment (***n*** = 36)		Control (***n*** = 36)	
	***n***	(%)	***n***	(%)	***n***	(%)
Gender						
Male	27	(38)	14	(39)	13	(36)
Female	45	(63)	22	(61)	23	(64)
Age (years), Mean ± SD	48.57 ± 14.80		49.42 ± 14.29		47.72 ± 15.45	
<40 years	18	(25)	10	(28)	8	(22)
40–65 years	46	(64)	22	(61)	24	(67)
>65 years	8	(11)	4	(11)	4	(11)
Underlying diseases						
Diabetes	17	(24)	11	(31)	6	(17)
Hypertension	29	(40)	16	(44)	13	(36)
Dyslipidemia	25	(35)	16	(44)	9	(25)
Ischemic heart disease	2	(3)	1	(3)	1	(3)
Peripheral arterial disease	0	(0)	0	(0)	0	(0)
Malignancy	0	(0)	0	(0)	0	(0)
HIV	0	(0)	0	(0)	0	(0)
Cerebrovascular disease	2	(3)	0	(0)	2	(6)
Alcoholism	0	(0)	0	(0)	0	(0)
Chronic liver disease	0	(0)	0	(0)	0	(0)
Chronic kidney disease stage	5	(7)	2	(6)	3	(8)
Others	29	(40)	13	(36)	16	(44)
Known mode of transmission	26	(36)	15	(42)	11	(31)
COVID-19 vaccine	48	(67)	23	(64)	25	(69)
1 dose	34	(47)	18	(50)	16	(44)
2 dose	13	(18)	5	(14)	8	(22)
Booster dose	1	(1)	0	(0)	1	(3)
AstraZeneca	41	(57)	20	(56)	21	(58)
Sinovac	3	(4)	2	(6)	1	(3)
Sinopharm	5	(7)	1	(3)	4	(11)
Time from last vaccine (days), median (IQR)	57	(34–66)	59	(38–70)	44	(11–64)

*Abbreviations*: *IQR* interquartile range

### Primary outcome

The proportion of patients in the treatment and control arm whose reverse transcription polymerase chain reaction (RT-PCR) result was negative day 7 (7 [17.3%] vs. 6 [14.3%], respectively; *p*=0.743) and day 14 (17 [47.2%] vs. 16 [44.4%], respectively; *p*=0.813) of enrollment did not significantly differ (Table 2). Furthermore, the Ct ratio day 14 also did not significantly differ between the treatment and control groups (17.43 ± 16.82 vs. 18.51 ± 17.34, respectively; *p*=0.788). One third of the patients in each group still had residual abnormal chest radiograph day 14 (12 [33%] vs. 11 [30.6%] in the treatment and control group; *p* = 0.800).

**Table 2:** Primary and secondary outcomes at days 7 and 14

Outcome/time	Treatment (***n*** = 36)	Control (***n*** = 36)	Absolute difference (95% CI)		Effect estimate (95% CI)	
Abnormal chest X-ray, No. (%)						
Day 1	11 (31%)	9 (25%)	0.06	(−0.15 to 0.26)^a^	1.22	(0.58 to 2.59)^c^
Day 14	12 (33%)	11 (31%)	0.03	(−0.19 to 0.24)^a^	1.09	(0.56 to 2.14)^c^
Positive PCR CT ratio, No. (%)						
Day 1	36 (100%)	36 (100%)	0.00	(0.00 to 0.00)^a^	1.00	(1.00 to 1.00)^c^
Day 7	29 (81%)	30 (83%)	−0.03	(−0.21 to 0.15)^a^	0.97	(0.78 to 1.20)^c^
Day 14	19 (53%)	20 (56%)	−0.03	(−0.26 to 0.20)^a^	0.95	(0.62 to 1.45)^c^
PCR CT ratio, Mean ± SD						
Day 1	23.65 ± 7.12	22.05 ± 5.10	1.60	(−1.31 to 4.51)^b^		
Day 7	25.99 ± 12.90	24.72 ± 11.17	1.27	(−4.48 to 7.03)^b^		
Day 14	17.43 ± 16.82	18.51 ± 17.34	−1.09	(−9.12 to 6.95)^b^		
N gene, Mean ± SD						
Day 1	23.36 ± 6.20	22.42 ± 5.46	0.93	(−1.81 to 3.68)^b^		
Day 7	28.62 ± 11.10	26.22 ± 10.60	2.21	(−3.04 to 7.45)^b^		
Day 14	20.02 ± 17.29	19.17 ± 17.09	0.85	(−7.23 to 0.93)^b^		

^a^Absolute difference is the difference in proportions

^b^Absolute difference is the mean difference

^c^Effect estimate is the risk ratios (RR)

### Clinical outcomes

**Fig. 2:**
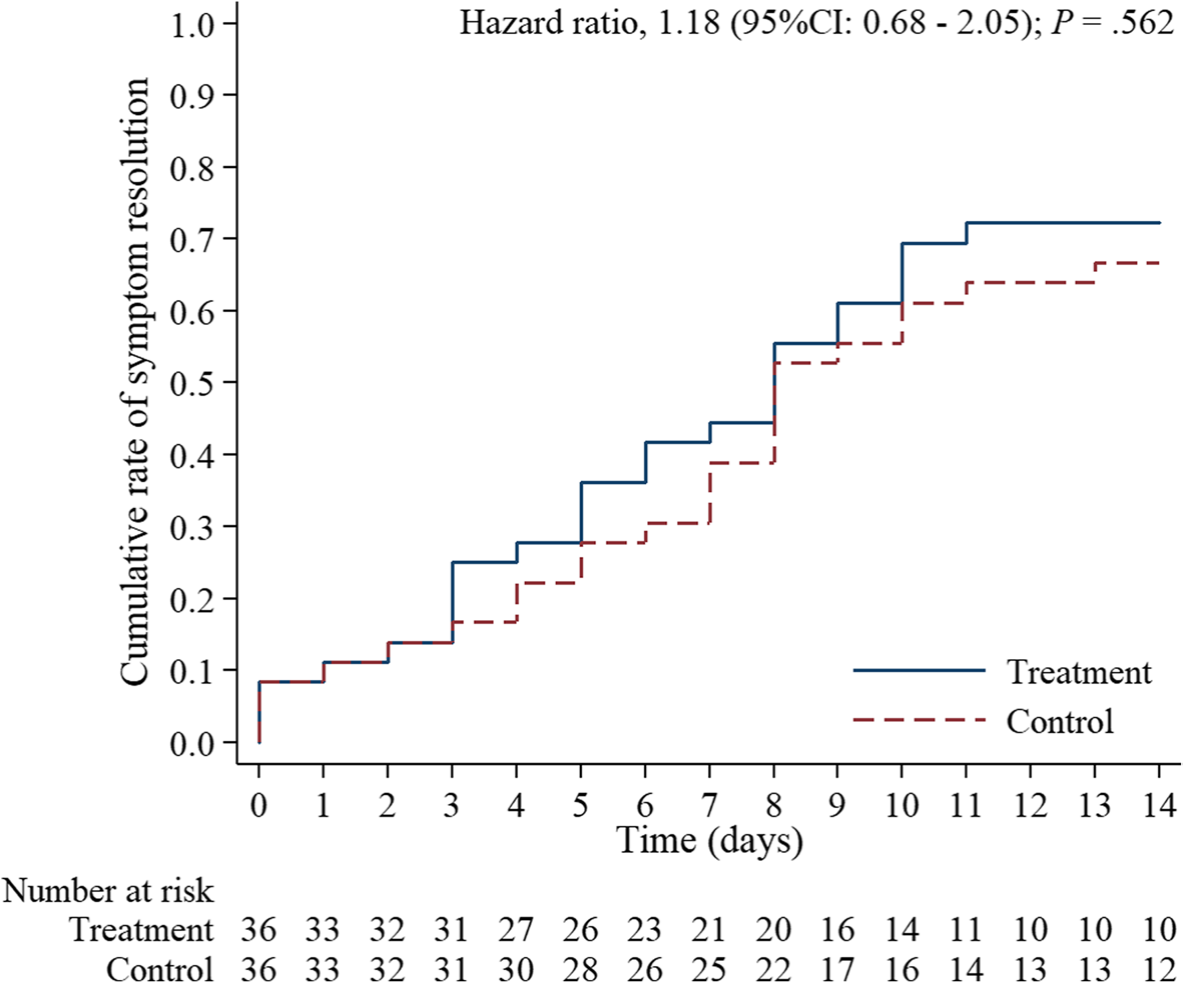
Kaplan–Meier survival analysis curve for time to resolution of symptoms in the ivermectin group (*n*=32) versus controls (*n*=32) (*p*=0.553)

The most common symptoms were fever (43.1%), cough (77.8%), and runny nose (50%), followed by loss of smell and taste (30.6 and 23.6%, respectively), sore throat (37.5%), and diarrhea (11%) (Table 3). Time to resolution of symptoms among patients assigned to ivermectin v.s placebo groups did not significantly differ (median, 8 days in both groups; HR for resolution of symptoms, 1.18 [95% CI, 0.68 to 2.65]; *p*=0.56). Table 4 shows the baseline and follow-up hemodynamics and vital signs from days 1 to 14. Both the control and treatment arms demonstrated stable blood pressure control, oxygen saturation, and respiratory rate throughout the disease course. None of the patients required intensive care unit admission or invasive ventilation. Nearly all of the patients were discharged by day 14, except two patients that requested discharge on day 10, and returned to repeat their laboratory tests day 14 on an outpatient basis. The hemodynamic characteristics did not significantly differ between the two groups from baseline until day 14 (Table 4). Time until resolution of symptoms among patients assigned to the ivermectin versus placebo group did not significantly differ between both groups (HR 1.18; 95% CI 0.67–2.08; *p*=0.572) (Fig. 2).

**Table 3:** Resolution of symptoms of COVID-19

Symptoms	Time to resolution of symptoms				Resolution of symptoms		
	Treatment (***n*** = 36)		Control (***n*** = 36)				
	Median	(95%CI)	Median	(95%CI)	HR^**a**^	95%CI	***p***-value
All symptoms	8	(5–10)	8	(7–13)	1.18	(0.68 to 2.05)	0.562
Cough	5	(3–8)	8	(4–8)	1.23	(0.74 to 2.03)	0.427
Runny nose	0	(0–3)	2	(0–5)	1.32	(0.82 to 2.13)	0.251
Sore throat	0	(0–3)	0	(0–3)	1.00	(0.63 to 1.59)	0.997
Smell disturbance	0	(0–0)	0	(0–0)	1.23	(0.76 to 1.99)	0.391
Taste disturbance	0	(0–1)	0	(0–0)	0.96	(0.60 to 1.53)	0.864
Muscle pain	0	(0–1)	0	(0–0)	0.81	(0.50 to 1.31)	0.388
Headache	0	(0–0)	0	(0–0)	1.25	(0.78 to 2.02)	0.354
Fever	0	(0–1)	0	(0–1)	0.90	(0.57 to 1.43)	0.650
Dyspnea	0	(0–0)	0	(0–0)	0.97	(0.61 to 1.54)	0.888
Block nose	0	(0–0)	0	(0–0)	1.08	(0.68 to 1.73)	0.733
Diarrhea	0	(0–0)	0	(0–0)	1.03	(0.65 to 1.65)	0.888
Chest pain	0	(0–0)	0	(0–0)	1.06	(0.66 to 1.69)	0.811
Fatigue	0	(0–0)	0	(0–0)	1.19	(0.73 to 1.92)	0.488
Sneezing	0	(0–0)	0	(0–0)	1.05	(0.66 to 1.67)	0.843
Vomiting	0	(0–0)	0	(0–0)	1.03	(0.65 to 1.64)	0.906

^a^Hazard ratio for resolution of symptoms was estimated by the Cox proportional-hazard model

**Table 4:** Evolution of hemodynamic status from day 1 to day 14

Outcomes	Day 1	Day 7	Day 14	Difference between groups^**b**^		
	Mean ± SD	Mean ± SD	Mean ± SD	Mean	95%CI	***p***-value
Temperature (°C)						
Treatment	36.54 ± 0.66	36.40 ± 0.35	36.54 ± 0.24	−0.06	(−0.16 to 0.05)	0.303
Control	36.65 ± 0.49	36.50 ± 0.30	36.48 ± 0.21		Reference	
***p*-value**^**a**^	0.441	0.176	0.584			
Heart rate (bpm)						
Treatment	90.86 ± 19.27	76.46 ± 14.82	76.43 ± 16.93	−0.04	(−14 to 0.07)	0.487
Control	90.63 ± 15.53	75.36 ± 11.66	74.83 ± 10.84		Reference	
***p*-value**^**a**^	0.956	0.730	0.804			
Systolic blood pressure (mmHg)						
Treatment	135.97 ± 25.54	118.11 ± 15.58	123.86 ± 10.57	1.58	(−3.76 to 6.91)	0.563
Control	129.69 ± 15.12	122.19 ± 16.30	121.67 ± 10.04		Reference	
***p*-value**^**a**^	0.221	0.285	0.658			
Diastolic blood pressure (mmHg)						
Treatment	79.49 ± 14.31	72.69 ± 10.32	76.29 ± 11.34	1.08	(−2.55 to 4.72)	0.559
Control	75.88 ± 12.77	70.50 ± 11.38	69.00 ± 8.29		Reference	
***p*-value**^**a**^	0.282	0.400	0.124			
Mean arterial pressure (mmHg)						
Treatment	98.31 ± 16.83	87.83 ± 9.97	92.14 ± 9.64	1.25	(−2.58 to 5.08)	0.523
Control	93.81 ± 11.39	87.73 ± 11.77	86.56 ± 7.67		Reference	
***p*-value**^**a**^	0.201	0.970	0.181			
Respiratory rate (bpm)						
Treatment	20.06 ± 0.58	19.94 ± 0.34	20.00 ± 0.00	0.03	(−0.08 to 0.13)	0.622
Control	19.94 ± 0.59	19.89 ± 0.67	19.67 ± 0.78		Reference	
***p*-value**^**a**^	0.421	0.670	0.166			
Oxygen saturation (%)						
Treatment	97.72 ± 1.23	97.67 ± 1.45	97.75 ± 1.59	−0.11	(−0.53 to 0.31)	0.605
Control	97.58 ± 1.46	97.75 ± 1.52	97.81 ± 1.21		Reference	
***p*-value**^**a**^	0.664	0.813	0.393			

^a^Comparison mean at point time between group using independent sample *t*-test

^b^Mean difference with 95% confidence interval estimated by linear mixed models

### Secondary outcome

All patients survived day 28 and almost all (92.1%) remained in the hospital until day 14. The proportion of patients who felt afebrile and healthy on day 14 did not significantly differ between the two treatment groups. The remaining symptoms upon discharge in the treatment and control arm were cough (19.4 and 19.4%), dyspnea (5.6 and 0%), smell disturbance (0 and 8.3%), runny nose (0.28 and 0%), sore throat (5.6 and 0%), headache (0 and 5.6%), muscle pain (8.3 and 2.8%), and malaise (0 and 5.6%). None of the patients required escalation of care. No major differences were found in the evolution of vital signs (Table 3), inflammatory markers (C-reactive protein, procalcitonin, ferritin, and interleukin-6), and other laboratory parameters among patients belonging to each group (Table 2S, Supplemental File). However, the time to resolution of many symptoms did not differ between both groups.

### Effect of vaccination

The proportion of patients receiving vaccination did not differ between the two groups, regardless of receiving one or two doses of a vaccine. Most patients were vaccinated with the ChAdOx1 nCoV-19 (Oxford-AstraZeneca) vaccine. Of the vaccinated patients, 47.2% received their first dose while only 18% received both doses of the vaccine (*p*=0.636). Time from last dose of vaccination to COVID-19 infection was comparable in both the ivermectin and control group.

### Adverse events

All patients completed the follow-up period of 28 days. No major adverse events were recorded among any patients during the trial period (14 days) and up to 28 days of follow-up. No major differences were observed in the evolution of vital signs, inflammatory markers (C-reactive protein, procalcitonin, and interleukin-6) and other laboratory parameters of patients in both groups (Supplemental File).

## Discussion

Even though effective vaccines and promising drugs for COVID-19 are now approved to emergency regulatory approval, efforts are ongoing to develop treatment options [16, 17]. Potential novel therapies are still extensively being reseached. Recently, the US Food and Drug Administration (FDA) has issued an emergency use authorization for Merck & Co.’s molnupiravir to treat mild to moderate COVID-19 among adults [18]. However, molnupiravir is not authorized for use among patients younger than 18 years of age, for the pre- or postexposure prevention of COVID-19, and also to treat hospitalized patients, due to its effect on bone and cartilage growth and the uncertainty of its efficacy when the treatment is initiated after hospitalization.

Ivermectin possesses antiparasitic and antiviral activities. Its efficacy has been shown in vitro against various viruses including dengue, Zika virus, West Nile virus, Venezuelan equine encephalitis virus, influenza virus, and SARS-CoV-2 [3, 19]. Since the start of the SARS-CoV-2 pandemic, both observational and randomized studies have evaluated ivermectin as a treatment for, and as prophylaxis against, COVID-19. A review by the Front Line COVID-19 Critical Care Alliance summarized findings from 27 studies on the effects of ivermectin to prevent and treat COVID-19, concluding that ivermectin “demonstrates a strong signal of therapeutic efficacy” against COVID 19 [6]. However, a recent meta-analysis did not reduce all-cause mortality, length of stay or viral clearance in RCTs among patients with mostly mild COVID-19 symptoms [20].

In the present study, a 5-day course of ivermectin did not improve clinical and microbiological outcomes of patients with mild or moderate COVID-19 symptoms. However, patients receiving ivermectin revealed a tendency to recover from certain symptoms earlier than those in the placebo group although without statistical significance. Several related studies reported more rapid viral clearance with the use of ivermectin [5, 7, 21]. However, other studies have not reported such a benefical outcome [22, 23]. However, some variation were noted in the regimes used by these studies. Although the effect on viral clearance remains unconfirmed, many studies have reported a significantly reduced time to recovery in the ivermectin group as compared with that of the control group [24–26]. Even when used to treat patients with severe COVID-19 symptoms, ivermectin can provide increased clinical recovery, improved prognostic laboratory parameters, and decreased mortality rates [27]. Moreover, vaccination did not affect viral clearance with the use of ivermectin in our study. This could be due to the incomplete vaccination status of the patients, i.e., a single dose received instead of the two-dose regime. Therefore, the patients might not have achieved sufficient neutralizing capacity.

Based on the results of the current study, we found that the time to clinical recovery did not significantly differ between ivermectin compared with the SOC. Symptoms such as runny nose, anosmia, fatique, and cough, which may indicate less progressive disease and rapid recovery, showed a tendency to recover sooner than those in the control group without reaching statistical significance. Ivermectin may help quicken the recovery by promoting faster viral clearance during disease onset, which might have prevented significant immune system involvement. In addition, early intervention rapidly reduced the viral load, thus preventing disease transmission in the general population. A larger randomized controlled clinical trial of ivermectin treatment is warranted to further validate these findings.

In the present study, we could not compare the length of hospital stay because the health policy in our country at the time of the study specified that every patient should be isolated for 14 days either in the hospital or at home. The effect of ivermectin on the length of hospital stay was therefore inconclusive. Bukhari et al. [28] randomized 86 patients with confirmed COVID-19 in SOC treatment and ivermectin (single dose of 12 mg) plus SOC treatment groups. They reported early viral clearance in the ivermectin group as compared with the SOC group (*p*=0.001). No adverse reactions were noted in the intervention arm. Ravikerti et al. reported that patients administered 12 mg ivermectin for 2 days showed no difference in the primary outcome, i.e., negative RT-PCR report day 6 of admission. However, a significantly higher proportion of patients were alive and discharged from the hospital when they received ivermectin [29]. Viral clearance was earlier in the 5-day ivermectin treatment arm when compared with the placebo group (9.7 days vs. 12.7 days, respectively, *p*=0.02) in the study by Ahmed [14]; however, the clearance of symptoms did not significantly differ between the two groups. Also, Chaccour et al. [25] found markedly reduced self-reported anosmia/hyposmia, reduced cough and a tendency to lower viral loads and lower IgG titers in a pilot, double-blind, placebo-controlled, single-center, parallel-arm, superiority, randomized clinical trial that compared a single dose of ivermectin with placebo among patients with nonsevere COVID-19 without any risk factors. These results provide evidence of the potential benefits of early intervention with ivermectin to treat mild to moderate COVID-19 symptoms.

This study encountered several limitations. First, the sample size was rather small and excluded patients with severe diseases or comorbidities. This was because the incidence of COVID-19 at the time of the study was rapidly decreasing in our country. We contained the pandemic quite well with low rates of new cases. Second, the duration of follow-up was short, i.e, up to 28 days only. A longer follow-up time might have revealed long-term benefits of ivermectin. Third, we included patients with mild to moderate COVID-19 symptoms, wherein the disease might subside spontaneaously without any proven benefit of any medications. Finally, the ivermectin dosage varied from study-to-study, and the exact and most appropriate dose of ivermectin remains unknown. Although in in vitro studies, the dose of ivermectin needed for inducing antiviral effects was higher than the usual dose approved among humans [25], high dose antiviral therapy could lead to severe adverse effects [30]. Further investigations are needed to adjust the proper dose of the medication to be approved as a COVID-19-specific treatment. The strength of this study was no patients were lost to follow-up, no missing data was observed because this study was conducted in only 14 days, all patients were in the hospital, and all data could be retrieved.

In conclusion, ivermectin did not significantly clear the virus and did not significantly improve the time to resolution of symptoms among patients with mild to moderate COVID-19 symptoms compared with SOC symptoms. However, a trend towards more resolution of some symptoms seemed to be higher in the ivermectin group. Therefore, given the urgent need to manage patients with COVID-19 using a safe, financially feasible and widely available drug, the present findings suggests that ivermectin can be considered as an add-on therapy to help modify the clinical course of COVID-19. A multicenter, double-blind, drug-controlled study will strengthen our findings.

## Supplementary Information

**Additional file 1.**

## Data Availability

Upon publication, all data supporting the results will be archived in a public repository accessible at Mendeley Data, V1, doi: 10.17632/ppg255h3bj.1.

## References

[1] COVID-19 dashboard. 2021. https://coronavirus.jhu.edu/map.html. Accessed 20 Nov 2021.

[2] Omura S, Crump A (2014). Ivermectin: panacea for resource-poor communities?. Trends Parasitol.

[3] Caly L, Druce JD, Catton MG, Jans DA, Wagstaff KM (2020). The FDA-approved drug ivermectin inhibits the replication of SARS-CoV-2 in vitro. Antiviral Res.

[4] Chen IS, Kubo Y (2018). Ivermectin and its target molecules: shared and unique modulation mechanisms of ion channels and receptors by ivermectin. J Physiol.

[5] Alam MT, Murshed R, Ebhiuyan E, Saber S, Alam RF, Robin RC (2020). A case series of 100 COVID-19 positive patients treated with combination of ivermectin and doxycycline. J Bangladesh Coll Phys.

[6] Kory P, GU M, Varon J, et al.. (2021). Review of the emerging evidence demonstrating the efficacy of ivermectin in the prophylaxis and treatment of COVID-19. Am J Ther.

[7] Khan MSI, Khan MSI, Debnath CR, Nath PN, Mahtab MA, Nabeka H (2020). Ivermectin treatment may improve the prognosis of patients with COVID-19. Arch Bronconeumol.

[8] Rajter JC, Sherman MS, Fatteh N, Vogel F, Sacks J, Rajter JJ (2021). Use of ivermectin is associated with lower mortality in hospitalized patients with coronavirus disease 2019: the ivermectin in COVID nineteen study. Chest.

[9] Formiga FR, Leblanc R, de Souza Rebouças J, Farias LP, de Oliveira RN, Pena L (2021). Ivermectin: an award-winning drug with expected antiviral activity against COVID-19. J Control Release.

[10] Bryant A, Lawrie TA, Dowswell T, Fordham EJ, Mitchell S, Hill SR, Tham TC (2021). Ivermectin for prevention and treatment of COVID-19 infection: a systematic review, meta-analysis, and trial sequential analysis to inform clinical guidelines. Am J Ther.

[11] Chachar AZK, Khan KA, Asif M, Tanveer K, Khaqan A, Basri R (2020). Effectiveness of ivermectin in SARS-CoV-2/COVID-19 patients. Int J Sci.

[12] Popp M, Stegemann M, Metzendorf MI, Gould S, Kranke P, Meybohm P, Skoetz N, Weibel S (2021). Ivermectin for preventing and treating COVID-19. Cochrane Database Syst Rev.

[13] Zu ZY, Jiang MD, Xu PP, Chen W, Ni QQ, Lu GM, Zhang LJ. Coronavirus Disease 2019 (COVID-19): A Perspective from China. Radiology. 2020;296(2):E15-E25. 10.1148/radiol.2020200490. 10.1148/radiol.2020200490 PMC7233368 32083985

[14] Bukhari KHS, Asghar A, Perveen N, Hayat A, Mangat SA, Kamil RB, et al. Efficacy of ivermectin in COVID-19 patients with mild to moderate disease. medRxiv 2021. 10.1101/2021.02.02.21250840 [Preprint 5 February 2021]. Ahmed S, Karim MM, Ross AG, Hossain MS, Clemens JD, Sumiya MK, et al. A five-day course of ivermectin for the treatment of COVID-19 may reduce the duration of illness. Int J Infect Dis. 2021;103:214–216. 10.1016/j.ijid.2020.11.191. 10.1016/j.ijid.2020.11.191 PMC7709596 33278625

[15] Kim J, Shin W (2014). How to do random allocation (randomization). Clin Orthop Surg.

[16] Galan LEB, Santos NMD, Asato MS, Araújo JV, de Lima Moreira A, Araújo AMM (2021). Phase 2 randomized study on chloroquine, hydroxychloroquine or ivermectin in hospitalized patients with severe manifestations of SARS-CoV-2 infection. Pathog Glob Health.

[17] Cortegiani A, Ippolito M, Greco M, Granone V, Protti A, Gregoretti C (2021). Rationale and evidence on the use of tocilizumab in COVID-19: a systematic review. Pulmonology.

[18] US Food and Drug Administration. https://www.fda.gov/news-events/press-announcements/coronavirus-covid-19-update-fda-authorizes-additional-oral-antiviral-treatment-covid-19-certain. Accessed 30 Nov 2021.

[19] Yang SNY, Atkinson SC, Wang C, Lee A, Bogoyevitch MA, Borg NA, Jans DA (2020). The broad spectrum antiviral ivermectin targets the host nuclear transport importin α/β1 heterodimer. Antiviral Res.

[20] Roman YM, Burela PA, Pasupuleti V, Piscoya A, Vidal JE, Hernandez AV. Ivermectin for the treatment of COVID-19: a systematic review and meta-analysis of randomized controlled trials. Clin Infect Dis. 2021;ciab591. 10.1093/cid/ciab591. 10.1093/cid/ciab591 PMC8394824 34181716

[21] Mahmud R, Rahman MM, Alam I, Ahmed KGU, Kabir AKMH, Sayeed SKJB (2021). Ivermectin in combination with doxycycline for treating COVID-19 symptoms: a randomized trial. J Int Med Res.

[22] Camprubí D, Almuedo-Riera A, Martí-Soler H, Soriano A, Hurtado JC, Subirà C (2020). Lack of efficacy of standard doses of ivermectin in severe COVID-19 patients. PLoS One.

[23] Choudhary R, Sharma AK (2020). Potential use of hydroxychloroquine, ivermectin and azithromycin drugs in fighting COVID-19: trends, scope and relevance. New Microbes New Infect.

[24] Hashim HA, et al. Controlled randomized clinical trial on using ivermectin with doxycycline for treating COVID-19 patients in Baghdad, Iraq. medRxiv. 2020. 10.1101/2020.10.26.20219345 [Preprint 27 October 2020].

[25] Chaccour C (2021). The effect of early treatment with ivermectin on viral load, symptoms and humoral response in patients with non-severe COVID-19: a pilot, double-blind, placebo-controlled, randomized clinical trial. EClinicalmedicine.

[26] Shahbaznejad L, Davoudi A, Eslami G, Markowitz J, Navaeifar M, Hosseinzadeh F (2021). Effects of ivermectin in patients with COVID-19: a multicenter, double-blind, randomized, controlled clinical trial. Clin Ther.

[27] Nurullah O, Demirtürk N, Çetinkaya R, Güner R, Avcı I, Orhan S (2021). Evaluation of the effectiveness and safety of adding ivermectin to treatment in severe COVID-19 patients. BMC Infect Dis.

[28] Ahmed S, Karim MM, Ross AG, Hossain MS, Clemens JD, Sumiya MK (2021). A five-day course of ivermectin for the treatment of COVID-19 may reduce the duration of illness. Int J Infect Dis.

[29] Ravikirti, Roy R, Pattadar C, Raj R, Agarwal N, Biswas B, et al. Ivermectin as a potential treatment for mild to moderate COVID-19 - a double blind randomized placebo-controlled trial. medRxiv. 2021. 10.1101/2021.01.05.21249310 [Preprint 9 January 2021]. 10.18433/jpps32105 34265236

[30] Heidary F, Gharebaghi R (2020). Ivermectin: a systematic review from antiviral effects to COVID-19 complementary regimen. J Antibiot (Tokyo).

