# Supplementary material for: Efficacy and safety of ivermectin in the treatment of mild to moderate COVID-19 infection: a randomized, double-blind, placebo-controlled trial
Source: Trials. 2022 Aug 26;23:714. doi: 10.1186/s13063-022-06649-3 (PMC9412770; doi:10.1186/s13063-022-06649-3)
Supplement: Supplementary file 1 — Additional file 1. [file 13063_2022_6649_MOESM1_ESM.docx]

| **Table 1S.** Laboratory investigation | | | | | | | | | | |  |
| --- | --- | --- | --- | --- | --- | --- | --- | --- | --- | --- | --- |
| **Variables** | **Day 1** | | | | | **Day 14** | | | | |  |
|  | **Treatment** | | **Control** | | **p-value^a^** | **Treatment** | | **Control** | | **p-value^a^** | |
| **Complete blood count** |  | |  |  |  |  |  |  |  |  | |
| Hemoglobin (g/dL) | 13.35 ± 1.35 | | 12.83 ± 1.58 | | 0.141 | 13.36 ± 1.60 | | 13.01 ± 1.66 | | 0.365 | |
| Hematocrit (%) | 40.60 ± 4.05 | | 39.15 ± 4.84 | | 0.174 | 40.16 ± 5.51 | | 39.85 ± 4.85 | | 0.798 | |
| White blood cell (X103/μl) | 5.94 ± 1.97 | | 6.74 ± 2.41 | | 0.128 | 8.17 ± 3.21 | | 9.37 ± 2.99 | | 0.106 | |
| Neutrophils (%) | 56.85 ± 7.72 | | 61.66 ± 13.6 | | 0.070 | 61.66 ± 8.79 | | 65.33 ± 8.97 | | 0.083 | |
| Lymphocytes (%) | 33.13 ± 7.64 | | 29.48 ± 12.14 | | 0.131 | 29.95 ± 7.90 | | 27.05 ± 8.27 | | 0.132 | |
| Monocytes (%) | 7.37 ± 2.53 | | 7.32 ± 3.00 | | 0.939 | 5.97 ± 1.64 | | 5.24 ± 1.24 | | 0.036 | |
| Platelets (X103/μl) | 232.25 ± 60.16 | | 252.92 ± 80.4 | | 0.221 | 330.31 ± 129.62 | | 352.58 ± 101.9 | | 0.420 | |
| **Blood chemistry panel** |  | | | | | | | | | |  |
| Blood urea nitrogen (mg/dL) | 10.39 ± 3.00 | | 10.94 ± 3.91 | | 0.501 | 14.36 ± 5.40 | | 14.67 ± 5.19 | | 0.807 | |
| Creatinine (mg/dL) | 0.83 ± 0.21 | | 0.85 ± 0.21 | | 0.675 | 0.85 ± 0.22 | | 0.78 ± 0.15 | | 0.136 | |
| Sodium (mEq/L) | 138.6 ± 3.01 | | 137.29 ± 3.91 | | 0.120 | 136.86 ± 3.20 | | 137.6 ± 2.92 | | 0.314 | |
| Potassium (mEq/L) | 3.75 ± 0.40 | | 3.81 ± 0.42 | | 0.535 | 4.02 ± 0.37 | | 4.02 ± 0.35 | | 0.971 | |
| Chloride (mEq/L) | 101.43 ± 3.08 | | 100.86 ± 3.38 | | 0.462 | 101.33 ± 3.70 | | 101.57 ± 3.59 | | 0.784 | |
| Bicarbonate (mEq/L) | 25.37 ± 2.37 | | 25.03 ± 2.88 | | 0.588 | 25.78 ± 2.95 | | 25.43 ± 2.80 | | 0.611 | |
| Total bilirubin (mg/dL) | 0.35 | (0.24 - 0.46) | 0.33 | (0.25 - 0.51) | 0.760 | 0.44 | (0.33 - 0.54) | 0.42 | (0.29 - 0.52) | 0.495 | |
| Direct bilirubin (mg/dL) | 0.18 | (0.12 - 0.25) | 0.175 | (0.14 - 0.22) | 0.824 | 0.23 | (0.15 - 0.25) | 0.21 | (0.15 - 0.25) | 0.709 | |
| SGOT  (U/L) | 31 | (25 - 39) | 30.5 | (23 - 40) | 0.901 | 25.5 | (20 - 33) | 26 | (19 - 30) | 0.685 | |
| SGPT  (U/L) | 23 | (18 - 33) | 23 | (15 - 34) | 0.879 | 32 | (14 - 50) | 22 | (16 - 48) | 0.481 | |
| Albumin (g/dL) | 4.30 ± 0.32 | | 4.14 ± 0.37 | | 0.067 | 4.10 ± 0.44 | | 4.07 ± 0.42 | | 0.798 | |
| Alkaline phosphatase (U/L) | 79.08 ± 20.67 | | 66.34 ± 23.51 | | 0.047 | 79.76 ± 22.83 | | 79.55 ± 21.18 | | 0.974 | |
| Total protein (g/dL) | 7.79 ± 0.49 | | 7.64 ± 0.47 | | 0.197 | 7.50 ± 0.69 | | 7.47 ± 0.70 | | 0.877 | |
| Fasting blood sugar (mg/dL) | 104 | (89 - 135) | 98 | (89 - 148) | 0.987 | 110 | (86 - 233) | 113 | (104 - 150) | 0.824 | |
| Dimer | 0.35 | (0.25 - 0.48) | 0.39 | (0.23 - 0.49) | 0.920 | 0.34 | (0.28 - 0.72) | 0.32 | (0.24 - 0.56) | 0.471 | |
| CRP | 10.85 | (3.45 - 20.15) | 12.25 | (4.72 - 24.93) | 0.398 | 1.69 | (0.64 - 6.38) | 2.46 | (1.08 - 4.63) | 0.548 | |
| Data are presented as mean ± standard deviation or median (interquartile range). | | | | | | | | | | | |
| P-value corresponds to Independent samples t-test or Mann-Whitney U test. | | | | | | | | | | | |

| **Table S2 Evolution of symptoms of COVID-19** | | | | | | | | | | | | | | | | | | |  | |  | |  | |  | |
| --- | --- | --- | --- | --- | --- | --- | --- | --- | --- | --- | --- | --- | --- | --- | --- | --- | --- | --- | --- | --- | --- | --- | --- | --- | --- | --- |
| **Symptoms** | **Group** | **Day 1** | **Day 2** | **Day 3** | **Day 4** | **Day 5** | **Day 6** | **Day 7** | **Day 8** | **Day 9** | **Day 10** | **Day 11** | **Day 12** | **Day 13** | **Day 14** | **Day 28** | **OR** | **95% CI** | | **p-value^b^** | | **OR_adj_** | | **95% CI** | | **p-value^c^** |
| All Symptoms | **Total** | 65 (90.3) | 65 (90.3) | 62 (86.1) | 57 (79.2) | 54 (75.0) | 48 (66.7) | 46 (63.9) | 42 (58.3) | 32 (44.4) | 29 (40.3) | 23 (31.9) | 25 (34.7) | 24 (33.3) | 24 (33.3) | 9 (12.5) |  |  | |  | |  | |  | |  |
|  | Treatment | 33 (91.7) | 32 (88.9) | 31 (86.1) | 27 (75.0) | 26 (72.2) | 22 (61.1) | 21 (58.3) | 20 (55.6) | 16 (44.4) | 14 (38.9) | 10 (27.8) | 11 (30.6) | 10 (27.8) | 11 (30.6) | 3 (8.3) | 0.63 | (0.16 - 2.59) | | 0.526 | | 0.52 | | (0.17 - 1.61) | | 0.255 |
|  | Control | 32 (88.9) | 33 (91.7) | 31 (86.1) | 30 (83.3) | 28 (77.8) | 26 (72.2) | 25 (69.4) | 22 (61.1) | 16 (44.4) | 15 (41.7) | 13 (36.1) | 14 (38.9) | 14 (38.9) | 13 (36.1) | 6 (16.7) | 1.00 | Reference | |  | | 1.00 | | Reference | |  |
|  | **p-value^a^** | 1.000 | 1.000 | 1.000 | 0.384 | 0.586 | 0.317 | 0.326 | 0.633 | 0.100 | 0.810 | 0.448 | 0.458 | 0.317 | 0.617 | 0.285 |  |  | |  | |  | |  | |  |
|  | **Total** | 56 (77.8) | 55 (76.4) | 53 (73.6) | 46 (63.9) | 42 (58.3) | 39 (54.2) | 36 (50.0) | 32 (44.4) | 26 (36.1) | 21 (29.2) | 17 (23.6) | 17 (23.6) | 17 (23.6) | 14 (19.4) | 5 (6.9) |  |  | |  | |  | |  | |  |
| Cough | Treatment | 26 (72.2) | 25 (69.4) | 24 (66.7) | 19 (52.8) | 19 (52.8) | 17 (47.2) | 15 (41.7) | 14 (38.9) | 13 (36.1) | 11 (30.6) | 8 (22.2) | 8 (22.2) | 8 (22.2) | 7 (19.4) | 2 (5.6) | 0.54 | (0.15 - 1.94) | | 0.346 | | 0.99 | | (0.39 - 2.55) | | 0.986 |
|  | Control | 30 (83.3) | 30 (83.3) | 29 (80.6) | 27 (75.0) | 23 (63.9) | 22 (61.1) | 21 (58.3) | 18 (50.0) | 13 (36.1) | 10 (27.8) | 9 (25.0) | 9 (25.0) | 9 (25.0) | 7 (19.4) | 3 (8.3) | 1.00 | Reference | |  | | 1.00 | | Reference | |  |
|  | **p-value^a^** | 0.275 | 0.165 | 0.181 | 0.050 | 0.339 | 0.237 | 0.157 | 0.343 | 1.000 | 0.795 | 0.781 | 0.781 | 0.781 | 1.000 | 1.000 |  |  | |  | |  | |  | |  |
|  | **Total** | 36 (50.0) | 30 (41.2) | 29 (40.3) | 29 (40.3) | 26 (36.1) | 23 (31.9) | 16 (22.2) | 12 (16.7) | 9 (12.5) | 8 (11.1) | 5 (6.9) | 6 (8.3) | 6 (8.3) | 4 (5.6) | 1 (1.4) |  |  | |  | |  | |  | |  |
| Runny nose | Treatment | 15 (41.7) | 14 (38.9) | 12 (33.3) | 12 (33.3) | 12 (33.3) | 11 (30.6) | 7 (19.4) | 6 (16.7) | 3 (8.3) | 3 (8.3) | 1 (2.8) | 1 (2.8) | 1 (2.8) | 0 (0.0) | 0 (0.0) | 0.37 | (0.08 - 1.67) | | 0.196 | | 0.95 | | (0.31 - 2.85) | | 0.921 |
|  | Control | 21 (58.3) | 16 (44.4) | 17 (47.2) | 17 (47.2) | 14 (38.9) | 12 (33.3) | 9 (25.0) | 6 (16.7) | 6 (16.7) | 5 (13.9) | 4 (11.1) | 5 (13.9) | 5 (13.9) | 4 (11.1) | 1 (2.8) | 1.00 | Reference | |  | | 1.00 | | Reference | |  |
|  | **p-value^a^** | 0.157 | 0.633 | 0.230 | 0.230 | 0.624 | 1.000 | 0.571 | 1.000 | 0.478 | 0.710 | 0.357 | 0.199 | 0.199 | 0.115 | 1.000 |  |  | |  | |  | |  | |  |
|  | **Total** | 27 (37.5) | 18 (25.0) | 26 (36.1) | 22 (30.6) | 21 (29.2) | 18 (25.0) | 11 (15.3) | 8 (11.1) | 8 (11.1) | 5 (6.9) | 3 (4.2) | 3 (4.2) | 2 (2.8) | 2 (2.8) | 0 (0.0) |  |  | |  | |  | |  | |  |
| Sore throat | Treatment | 14 (38.9) | 9 (25.0) | 13 (36.1) | 11 (30.6) | 11 (30.6) | 8 (22.2) | 5 (13.9) | 5 (13.9) | 4 (11.1) | 2 (5.6) | 1 (2.8) | 2 (5.6) | 1 (2.8) | 2 (5.6) | 0 (0.0) | 1.18 | (0.23 - 6.20) | | 0.844 | | 0.99 | | (0.37 - 2.65) | | 0.978 |
|  | Control | 13 (36.1) | 9 (25.0) | 13 (36.1) | 11 (30.6) | 10 (27.8) | 10 (27.8) | 6 (16.7) | 3 (8.3) | 4 (11.1) | 3 (8.3) | 2 (5.6) | 1 (2.8) | 1 (2.8) | 0 (0.0) | 0 (0.0) | 1.00 | Reference | |  | | 1.00 | | Reference | |  |
|  | **p-value^a^** | 0.808 | 1.000 | 1.000 | 1.000 | 0.759 | 0.589 | 0.743 | 0.710 | 1.000 | 1.000 | 1.000 | 1.000 | 1.000 | 0.493 | NA |  |  | |  | |  | |  | |  |
|  | **Total** | 22 (30.6) | 15 (20.8) | 21 (29.2) | 18 (25.0) | 10 (13.9) | 7 (9.7) | 6 (8.3) | 5 (9.4) | 4 (5.6) | 4 (5.6) | 4 (5.6) | 4 (5.6) | 3 (4.2) | 3 (4.2) | 2 (2.8) |  |  | |  | |  | |  | |  |
| Smell disturbance | Treatment | 10 (27.8) | 6 (16.7) | 9 (25.0) | 9 (25.0) | 4 (11.1) | 3 (8.3) | 2 (5.6) | 1 (2.8) | 0 (0.0) | 0 (0.0) | 0 (0.0) | 0 (0.0) | 0 (0.0) | 0 (0.0) | 0 (0.0) | 0.34 | (0.04 - 3.11) | | 0.342 | | 0.52 | | (0.15 - 1.80) | | 0.305 |
|  | Control | 12 (33.3) | 9 (25.0) | 12 (33.3) | 9 (25.0) | 6 (16.7) | 4 (11.1) | 4 (11.1) | 4 (11.1) | 4 (11.1) | 4 (11.1) | 4 (11.1) | 4 (11.1) | 3 (8.3) | 3 (8.3) | 2 (5.6) | 1.00 | Reference | |  | | 1.00 | | Reference | |  |
|  | **p-value^a^** | 0.609 | 0.384 | 0.437 | 1.000 | 0.496 | 1.000 | 0.674 | 0.357 | 0.115 | 0.115 | 0.115 | 0.115 | 0.239 | 0.239 | 0.493 |  |  | |  | |  | |  | |  |
|  | **Total** | 17 (23.6) | 15 (20.8) | 12 (16.7) | 12 (16.7) | 11 (15.3) | 9 (12.5) | 7 (9.7) | 4 (5.6) | 2 (2.8) | 1 (1.4) | 1 (1.4) | 1 (1.4) | 0 (0.0) | 1 (1.4) | 0 (0.0) |  |  | |  | |  | |  | |  |
| Taste disturbance | Treatment | 12 (33.3) | 10 (27.8) | 7 (19.4) | 7 (19.4) | 5 (13.9) | 4 (11.1) | 4 (11.1) | 2 (5.6) | 1 (2.8) | 0 (0.0) | 0 (0.0) | 0 (0.0) | 0 (0.0) | 1 (2.8) | 0 (0.0) | 4.81 | (0.53 - 43.79) | | 0.163 | | 0.53 | | (0.14 - 2.02) | | 0.353 |
|  | Control | 5 (13.9) | 5 (13.9) | 5 (13.9) | 5 (13.9) | 6 (16.7) | 5 (13.9) | 3 (8.3) | 2 (5.6) | 1 (2.8) | 1 (2.8) | 1 (2.8) | 1 (2.8) | 0 (0.0) | 0 (0.0) | 0 (0.0) | 1.00 | Reference | |  | | 1.00 | | Reference | |  |
|  | **p-value^a^** | 0.052 | 0.147 | 0.527 | 0.527 | 0.743 | 1.000 | 1.000 | 1.000 | 1.000 | 1.000 | 1.000 | 1.000 | NA | 1.000 | NA |  |  | |  | |  | |  | |  |
|  | **Total** | 15 (20.8) | 16 (22.2) | 12 (16.7) | 10 (13.9) | 7 (9.7) | 6 (8.3) | 4 (5.6) | 3 (4.2) | 3 (4.2) | 4 (5.6) | 3 (4.2) | 3 (4.2) | 3 (4.2) | 4 (5.6) | 2 (2.8) |  |  | |  | |  | |  | |  |
| Muscle pain | Treatment | 10 (27.8) | 10 (27.8) | 7 (19.4) | 6 (16.7) | 5 (13.9) | 4 (11.1) | 3 (8.3) | 3 (8.3) | 3 (8.3) | 2 (5.6) | 2 (5.6) | 2 (5.6) | 2 (5.6) | 3 (8.3) | 1 (2.8) | 3.84 | (0.56 - 26.37) | | 0.171 | | 1.23 | | (0.27 - 5.60) | | 0.785 |
|  | Control | 5 (13.9) | 6 (16.7) | 5 (13.9) | 4 (11.1) | 2 (5.6) | 2 (5.6) | 1 (2.8) | 0 (0.0) | 0 (0.0) | 2 (5.6) | 1 (2.8) | 1 (2.8) | 1 (2.8) | 1 (2.8) | 1 (2.8) | 1.00 | Reference | |  | | 1.00 | | Reference | |  |
|  | **p-value^a^** | 0.147 | 0.257 | 0.527 | 0.496 | 0.429 | 0.674 | 0.614 | 0.239 | 0.239 | 1.000 | 1.000 | 1.000 | 1.000 | 0.614 | 1.000 |  |  | |  | |  | |  | |  |
|  | **Total** | 15 (20.8) | 18 (25.0) | 12 (16.7) | 11 (15.3) | 6 (8.3) | 3 (4.7) | 3 (4.7) | 4 (5.6) | 3 (4.2) | 2 (2.8) | 2 (2.8) | 2 (2.8) | 2 (2.8) | 2 (2.8) | 0 (0.0) |  |  | |  | |  | |  | |  |
| Headache | Treatment | 5 (13.9) | 8 (22.2) | 6 (16.7) | 4 (11.1) | 2 (5.6) | 0 (0.0) | 0 (0.0) | 0 (0.0) | 0 (0.0) | 0 (0.0) | 0 (0.0) | 0 (0.0) | 0 (0.0) | 0 (0.0) | 0 (0.0) | 0.24 | (0.03 - 1.71) | | 0.153 | | 0.80 | | (0.20 - 3.19) | | 0.747 |
|  | Control | 10 (27.8) | 10 (27.8) | 6 (16.7) | 7 (19.4) | 4 (11.1) | 3 (8.3) | 3 (8.3) | 4 (11.1) | 3 (8.3) | 2 (5.6) | 2 (5.6) | 2 (5.6) | 2 (5.6) | 2 (5.6) | 0 (0.0) | 1.00 | Reference | |  | | 1.00 | | Reference | |  |
|  | **p-value^a^** | 0.147 | 0.586 | 1.000 | 0.326 | 0.674 | 0.293 | 0.239 | 0.115 | 0.239 | 0.493 | 0.493 | 0.493 | 0.493 | 0.493 | NA |  |  | |  | |  | |  | |  |
|  | **Total** | 31 (43.1) | 12 (16.7) | 11 (15.3) | 8 (11.1) | 3 (4.2) | 3 (4.2) | 0 (0.0) | 0 (0.0) | 0 (0.0) | 0 (0.0) | 0 (0.0) | 0 (0.0) | 0 (0.0) | 0 (0.0) | 0 (0.0) |  |  | |  | |  | |  | |  |
| Fever | Treatment | 16 (44.4) | 7 (19.4) | 7 (19.4) | 5 (13.9) | 2 (5.6) | 1 (2.8) | 0 (0.0) | 0 (0.0) | 0 (0.0) | 0 (0.0) | 0 (0.0) | 0 (0.0) | 0 (0.0) | 0 (0.0) | 0 (0.0) | 1.33 | (0.61 - 2.91) | | 0.468 | | 1.25 | | (0.72 - 2.16) | | 0.424 |
|  | Control | 15 (41.7) | 5 (13.9) | 4 (11.1) | 3 (8.3) | 1 (2.8) | 2 (5.6) | 0 (0.0) | 0 (0.0) | 0 (0.0) | 0 (0.0) | 0 (0.0) | 0 (0.0) | 0 (0.0) | 0 (0.0) | 0 (0.0) | 1.00 | Reference | |  | | 1.00 | | Reference | |  |
|  | **p-value^a^** | 0.812 | 0.527 | 0.326 | 1.000 | 1.000 | 1.000 | NA | NA | NA | NA | NA | NA | NA | NA | NA |  |  | |  | |  | |  | |  |
|  | **Total** | 12 (16.7) | 7 (9.7) | 6 (8.3) | 4 (4.6) | 5 (6.9) | 6 (8.3) | 5 (6.9) | 3 (4.2) | 5 (6.9) | 4 (5.6) | 3 (4.2) | 3 (4.2) | 3 (4.2) | 2 (2.8) | 1 (1.4) |  |  | |  | |  | |  | |  |
| Dyspnea | Treatment | 5 (13.9) | 3 (8.3) | 3 (8.3) | 2 (5.6) | 4 (11.1) | 4 (11.1) | 3 (8.3) | 2 (5.6) | 3 (8.3) | 2 (5.6) | 2 (5.6) | 2 (5.6) | 2 (5.6) | 2 (5.6) | 1 (2.8) | 1.09 | (0.15 - 7.96) | | 0.934 | | 1.96 | | (0.29 - 13.30) | | 0.493 |
|  | Control | 7 (19.4) | 4 (11.1) | 3 (8.3) | 2 (5.6) | 1 (2.8) | 2 (5.6) | 2 (5.6) | 1 (2.8) | 2 (5.6) | 2 (5.6) | 1 (2.8) | 1 (2.8) | 1 (2.8) | 0 (0.0) | 0 (0.0) | 1.00 | Reference | |  | | 1.00 | | Reference | |  |
|  | **p-value^a^** | 0.527 | 1.000 | 1.000 | 0.710 | 0.357 | 0.674 | 1.000 | 1.000 | 1.000 | 1.000 | 1.000 | 1.000 | 1.000 | 0.493 | 1.000 |  |  | |  | |  | |  | |  |
|  | **Total** | 9 (12.5) | 9 (12.5) | 9 (12.5) | 6 (8.3) | 2 (2.8) | 0 (0.0) | 1 (1.4) | 1 (1.4) | 1 (1.4) | 1 (1.4) | 1 (1.4) | 1 (1.4) | 1 (1.4) | 2 (2.8) | 0 (0.0) |  |  | |  | |  | |  | |  |
| Runny nose | Treatment | 4 (11.1) | 4 (11.1) | 4 (11.1) | 2 (5.6) | 1 (2.8) | 0 (0.0) | 0 (0.0) | 0 (0.0) | 0 (0.0) | 0 (0.0) | 0 (0.0) | 0 (0.0) | 0 (0.0) | 0 (0.0) | 0 (0.0) | 0.51 | (0.04 - 6.53) | | 0.604 | | 0.46 | | (0.14 - 1.60) | | 0.223 |
|  | Control | 5 (13.9) | 5 (13.9) | 5 (13.9) | 4 (11.1) | 1 (2.8) | 0 (0.0) | 1 (2.8) | 1 (2.8) | 1 (2.8) | 1 (2.8) | 1 (2.8) | 1 (2.8) | 1 (2.8) | 2 (5.6) | 0 (0.0) | 1.00 | Reference | |  | | 1.00 | | Reference | |  |
|  | **p-value^a^** | 1.000 | 1.000 | 1.000 | 0.674 | 1.000 | NA | 1.000 | 1.000 | 1.000 | 1.000 | 1.000 | 1.000 | 1.000 | 0.493 | NA |  |  | |  | |  | |  | |  |
|  | **Total** | 8 (11.10 | 3 (4.2) | 1 (1.4) | 3 (4.2) | 3 (4.2) | 0 (0.0) | 0 (0.0) | 0 (0.0) | 1 (1.4) | 0 (0.0) | 0 (0.0) | 0 (0.0) | 0 (0.0) | 0 (0.0) | 0 (0.0) |  |  | |  | |  | |  | |  |
| Diarrhea | Treatment | 4 (11.1) | 0 (0.0) | 0 (0.0) | 2 (5.6) | 2 (5.6) | 0 (0.0) | 0 (0.0) | 0 (0.0) | 1 (2.8) | 0 (0.0) | 0 (0.0) | 0 (0.0) | 0 (0.0) | 0 (0.0) | 0 (0.0) | 0.97 | (0.28 - 3.34) | | 0.958 | | 0.89 | | (0.35 - 2.27) | | 0.812 |
|  | Control | 4 (11.1) | 3 (8.3) | 1 (2.8) | 1 (2.8) | 1 (2.8) | 0 (0.0) | 0 (0.0) | 0 (0.0) | 0 (0.0) | 0 (0.0) | 0 (0.0) | 0 (0.0) | 0 (0.0) | 0 (0.0) | 0 (0.0) | 1.00 | Reference | |  | | 1.00 | | Reference | |  |
|  | **p-value^a^** | 1.000 | 0.293 | 1.000 | 1.000 | 1.000 | NA | NA | NA | 1.000 | NA | NA | NA | NA | NA | NA |  |  | |  | |  | |  | |  |
|  | **Total** | 1 (1.4) | 1 (1.4) | 1 (1.4) | 1 (1.4) | 2 (2.8) | 2 (2.8) | 2 (2.8) | 2 (2.8) | 0 (0.0) | 0 (0.0) | 0 (0.0) | 0 (0.0) | 0 (0.0) | 0 (0.0) | 0 (0.0) |  |  | |  | |  | |  | |  |
| Chest pain | Treatment | 0 (0.0) | 0 (0.0) | 0 (0.0) | 0 (0.0) | 0 (0.0) | 0 (0.0) | 0 (0.0) | 0 (0.0) | 0 (0.0) | 0 (0.0) | 0 (0.0) | 0 (0.0) | 0 (0.0) | 0 (0.0) | 0 (0.0) | - | - | | NA | |  | |  | |  |
|  | Control | 1 (2.8) | 1 (2.8) | 1 (2.8) | 1 (2.8) | 2 (5.6) | 2 (5.6) | 2 (5.6) | 2 (5.6) | 0 (0.0) | 0 (0.0) | 0 (0.0) | 0 (0.0) | 0 (0.0) | 0 (0.0) | 0 (0.0) | 1.00 | Reference | |  | |  | |  | |  |
|  | **p-value^c^** | 1.000 | 1.000 | 1.000 | 1.000 | 0.493 | 0.493 | 0.493 | 0.493 | NA | NA | NA | NA | NA | NA | NA |  |  | |  | |  | |  | |  |
|  | **Total** | 3 (4.2) | 2 (2.8) | 3 (4.2) | 2 (2.8) | 3 (4.2) | 2 (2.8) | 2 (2.8) | 2 (2.8) | 2 (2.8) | 2 (2.8) | 2 (2.8) | 3 (4.2) | 2 (2.8) | 2 (2.8) | 0 (0.0) |  |  | |  | |  | |  | |  |
| Fatique | Treatment | 0 (0.0) | 0 (0.0) | 0 (0.0) | 0 (0.0) | 0 (0.0) | 0 (0.0) | 0 (0.0) | 0 (0.0) | 0 (0.0) | 0 (0.0) | 0 (0.0) | 1 (2.8) | 0 (0.0) | 0 (0.0) | 0 (0.0) | 0.13 | (0.00 - 8.94) | | 0.345 | |  | |  | |  |
|  | Control | 3 (8.3) | 2 (5.6) | 3 (8.3) | 2 (5.6) | 3 (8.3) | 2 (5.6) | 2 (5.6) | 2 (5.6) | 2 (5.6) | 2 (5.6) | 2 (5.6) | 2 (5.6) | 2 (5.6) | 2 (5.6) | 0 (0.0) | 1.00 | Reference | |  | |  | |  | |  |
|  | **p-value^a^** | 0.239 | 0.439 | 0.239 | 0.493 | 0.239 | 0.493 | 0.493 | 0.439 | 0.493 | 0.493 | 0.493 | 1.000 | 0.493 | 0.493 | NA |  |  | |  | |  | |  | |  |
|  | **Total** | 1 (1.4) | 1 (1.4) | 1 (1.4) | 1 (1.4) | 0 (0.0) | 1 (1.4) | 0 (0.0) | 0 (0.0) | 0 (0.0) | 0 (0.0) | 0 (0.0) | 0 (0.0) | 0 (0.0) | 0 (0.0) | 0 (0.0) |  |  | |  | |  | |  | |  |
| Sneezing | Treatment | 0 (0.0) | 0 (0.0) | 0 (0.0) | 0 (0.0) | 0 (0.0) | 1 (2.8) | 0 (0.0) | 0 (0.0) | 0 (0.0) | 0 (0.0) | 0 (0.0) | 0 (0.0) | 0 (0.0) | 0 (0.0) | 0 (0.0) | 0.22 | (0.00 - 12.54) | | 0.465 | |  | |  | |  |
|  | Control | 1 (2.8) | 1 (2.8) | 1 (2.8) | 1 (2.8) | 0 (0.0) | 0 (0.0) | 0 (0.0) | 0 (0.0) | 0 (0.0) | 0 (0.0) | 0 (0.0) | 0 (0.0) | 0 (0.0) | 0 (0.0) | 0 (0.0) | 1.00 | Reference | |  | |  | |  | |  |
|  | **p-value^a^** | 1.000 | 1.000 | 1.000 | 1.000 | NA | 1.000 | NA | NA | NA | NA | NA | NA | NA | NA | NA |  |  | |  | |  | |  | |  |
|  | **Total** | 0 (0.0) | 1 (1.4) | 0 (0.0) | 0 (0.0) | 0 (0.0) | 0 (0.0) | 0 (0.0) | 0 (0.0) | 0 (0.0) | 0 (0.0) | 0 (0.0) | 0 (0.0) | 0 (0.0) | 0 (0.0) | 0 (0.0) |  |  | |  | |  | |  | |  |
| Vomitting | Treatment | 0 (0.0) | 0 (0.0) | 0 (0.0) | 0 (0.0) | 0 (0.0) | 0 (0.0) | 0 (0.0) | 0 (0.0) | 0 (0.0) | 0 (0.0) | 0 (0.0) | 0 (0.0) | 0 (0.0) | 0 (0.0) | 0 (0.0) | - | - | | NA | |  | |  | |  |
|  | Control | 0 (0.0) | 1 (2.8) | 0 (0.0) | 0 (0.0) | 0 (0.0) | 0 (0.0) | 0 (0.0) | 0 (0.0) | 0 (0.0) | 0 (0.0) | 0 (0.0) | 0 (0.0) | 0 (0.0) | 0 (0.0) | 0 (0.0) | 1.00 | Reference | |  | |  | |  | |  |
|  | **p-value^a^** | NA | 1.000 | NA | NA | NA | NA | NA | NA | NA | NA | NA | NA | NA | NA | NA |  |  | |  | |  | |  | |  |
|  |  |  |  |  |  |  |  |  |  |  |  |  |  |  |  |  |  |  | |  | |  | |  | |  |
| 1. p-value from Chi-square test or Fisher’s exact test; b) p-value from Multilevel mixed-effects logistic regression analysis; c) p-value from Multilevel mixed-effects logistic regression analysis adjusted for base line (day 1). | | | | | | | | | | | | | | | | | | | | | | | | | | |

**Study Protocol**

1. ***Objective***

**Main objective**

To study the efficacy of ivermectin as an addition to the standard of care treatment in COVID-19 patients with mild and moderate disease. mainly the virus SARS-Co-V 2 clearance rate at day 7 and day 14.

**Secondary objectives**

- To compare all-cause mortality rate between using ivermectin and standard of care treatment.

- To evaluate the complications after using ivermectin.

- To study factors that associated with clinical and laboratory recovery such as need for oxygen support , the duration of hospitalization and C-reactive protein .

1. **Conceptual framework**

**CONSORT 2010 Flow Diagram**

Excluded

Analysed

## Follow-Up

Analysed

## Analysis

Follow the viral clearance rate of SARS-Co-V 2 using RT-PCR test and complications after using ivermectin for 14 days.

Follow the viral clearance rate of SARS-Co-V 2 using RT-PCR test and complications after using ivermectin for 14 days.

## Enrollment

Allocated to ivermectin

CBC, BUN, Electrolyte, Ca, PO4, LFT at admission

## Allocation

Allocated to standard of care

CBC, BUN, Electrolyte, Ca, PO4, LFT at admission

Randomized

Assessed for eligibility

**Methodology**

**Research pattern**

Randomized , double-blinded , placebo-controlled,single-center,parallel-arm,superiority trial trial.

1. The COVID-19 patients who were confirmed by RT-PCR test and received the treatment at Vajira Hospital

2. Compare with the group of patients who receive standard of care.

**Population**

Inclusion criteria

1. Patients diagnosed with COVID-19 and confirmed with a positive SARS-CoV-2 PCR.

2. The patient should be between the ages of 18 and 80 years of age.

3. Negative pregnancy test for women of child bearing age.

4. The patient or his/her representative, have given consent to participate in the study.

5. Do or do not have these symptoms: fever (more than 37.5 ° c), cough and sore throat.

6. Have mild or moderate illness which have following the symptoms, temperature of more than 37.5 ° c, cough, sore throat, loss of smell, loss of taste, red eyes, rashes, diarrhea but there are no signs of tachypnea or dyspnea, pneumonia, no risk factors or major comorbidities.

The moderate illness patients have the risk factors for severe illness. They will have chest pain and shortness of breath while doing activities, rapid breathing or trouble breathing, pneumonia but the oxygen saturation is still more than 90% and have diarrhea more than 3 times a day.

**Exclusion criteria**

1. Known history of Ivermectin allergy.

2. COVID-19 Pneumonia.

3. Fever or cough present for more than 72 hours.

4. Age under 18 or over 60 years.

5. The following co-morbidities (or any other disease that might interfere with the study in the eyes of the investigator):

• Immunosuppression

• Chronic Obstructive Pulmonary Disease

• Acute or chronic renal failure

• History of coronary disease

• History of cerebrovascular disease

• Current neoplasm

6. Current use of CYP 3A4 or P-gp inhibitor drugs such as quinidine, amiodarone, diltiazem, spironolactone, verapamil, clarithromycin, erythromycin, itraconazole, ketoconazole, cyclosporine, tacrolimus, indinavir, ritonavir or cobicistat. Use of critical CYP3A4 substrate drugs such as warfarin.

7. History of ivermectin treatment in previous 7 days.

8. Patient refused to participate in the study.

**Termination criteria**

Patients will be informed that they may be withdrawn from the study at any time without providing any explanation. Withdrawal may also take place for any of the following reasons:

- Loss of monitoring

The investigator shall strive to ensure that the patient agrees to the final trial visit. The data of the withdrawn patients shall be collected up to this final visit. If the withdrawal is the outcome of a Serious adverse event related to ivermectin or SARS-CoV-2, the data shall be collected until the patient recovers or stabilizes.

- There are unusual symptoms during the research such as allergy, rashes or others.

The standard treatment is favipiravir,corticosteroids,andrographolide,lopinavir/ritonavir

The duration of infection starts from the day that patients show the symptoms.

**Inform consent process and recruitment process**

The research coordinator will inform all the detail to the patients either in the emergency room or by using a cellphone or VDO call.

- Whenever the patients sign consents then they will be randomized by the pharmacist in to intervention and control group .

**Methodology**

- The patients will be examined by the general practitioner and performed the laboratory tests which included: complete blood count, blood urea nitrogen, creatinine, liver function test, blood sugar, C-reactive protein ,chest x-ray and ECG.

- Repeat the blood tests again on day 14.

- Randomization process

By using systemic random sampling by providing 2 letters “A” envelops, which means the patients received ivermectin, and 2 letter “B” envelops, which means the patients received standard drug. One envelop is for 4 patients. There will be 5 envelops (20 patients). The envelops will be in a random order, for example, the first envelop is AABB, the second envelope is ABAB, the third envelope is BABA, etc.

3. Randomly dividing the patients into 2 groups.

- Group 1: receiving 12 mg. of ivermectin for 5 days along with a standard therapy.

- Group 2: receiving the standard drug, favipiravir or others.

- Follow up on swab RT-PCR test of COVID-19 on day 7, 14

- Record the total days of hospitalization

-The research coordinator and nurses will record the symptoms every day by telephone

- The patients will stay in this research project at least 14 days if there are no complications. However, the patients might be in this project longer if complications occur.

**-** If the patients have more severe illness, the patients will be treated according the guidlineline such as moving to the hospital or the intensive care unit

- There will be daily follow- up on patient’s symptoms including the side-effect.

The measuring variables include

- RT-PCR test results

- All-cause mortality rate.

- The drug’s side-effect.

**1. Introduction**

The emergent outbreak of coronavirus disease (COVID-19), caused by severe acute respiratory syndrome coronavirus 2 (SARS-CoV-2), has emphasized the requirement for therapeutic opportunities to overcome this pandemic. Ivermectin is an antiparasitic drug that has shown effectiveness against various agents, including SARS-CoV-2. This study aimed to assess the efficacy of ivermectin treatment compared with the standard of care among people with mild to moderate COVID-19.

**2. Study design**

A randomized, double-blind, placebo-controlled, single-center, parallel-arm, superiority trial

2.1 Sample size calculation

The sample size of 25 per group was based on the comparison of two proportions and calculated to have 90% power at a two-sided significance level of 0.05, allocation ratio 1:1 with continuity calculation using Stata, Version 16.0 to detect a 45.8% reduction (90.2 vs 44.4%) in the proportion of participants with positive PCR at day 7 posttreatment (Efficacy of Ivermectin among patients with COVID-19 with mild to moderate disease showed a proportion with positive PCR at day 7 in intervention and control groups 9.8% (4/41; p1 = 0.098) and 55.6% (25/45; p2 = 0.556)), respectively. The sample size will be inflated to 36 participants per group (72 in total) to account for a possible 30% loss-to-follow-up, noncompliance and drop-out.

**3. Aims and objectives**

This study aimed to assess the efficacy of ivermectin treatment compared with the standard of care among people with mild to moderate COVID-19.

**4. Outcomes**

4.1 Primary outcome

The negativity of reverse transcription polymerase chain reaction (RT-PCR) results at day 7 and 14 of enrollment

4.2 Secondary outcomes

Resolution of symptoms of COVID-19 including cough, runny nose, sore throat, smell disturbance, taste disturbance, muscle pain, headache, fever, dyspnea, block nose, diarrhea, chest pain, fatigue, sneezing and vomiting.

Evolution of hemodynamic status from days 1 to 14 including temperature, heart rate, systolic blood pressure, diastolic blood pressure, mean arterial pressure, respiratory rate and oxygen saturation.

4.3 Safety outcome

To study the side effects of ivermectin in addition to standard treatment for patients with COVID-19.

**5. Populations**

**Intention-to-treat (ITT)**

All randomized subjects will be considered as the primary population for the analysis.

**Per Protocol (PP)**

All randomized study subjects completing the whole study period (complete cases) will be included. For a specific analysis, study subjects with protocol deviation/noncompliance and missing data on any of the variables in the model will be excluded from the analysis. Analyses of this population are seen as a sensitivity assessment to investigate whether findings are sensitive to assumptions regarding the pattern of missing data.

**6. Analyses**

Descriptive statistics will be used for demographic and clinical data. Variables will be presented as means and standard deviation for continuous data and as the frequency and percentages for categorical data. Continuous data will be expressed as mean ± standard deviation and median (25th and 75th percentiles) for normally distributed and nonnormally distributed data, respectively. Statistical significance is set at P <.05, and all tests were two-tailed. Statistical analysis will be performed using Stata, Version 16.0 (StataCorp).

6.1 Primary outcome

The primary objective is the proportion of participants with positive PCR at day 7 and 14 posttreatment. Proportions are compared between ivermectin and placebo using Fisher’s exact test and presented as a risk ratio (RR) with 95% confidence interval (CI). Mean PCR CT ratio and N gene will be compared between ivermectin and placebo using Student’s t-test and presented as a mean difference with 95% CI.

6.2 Secondary outcomes

Resolution of symptoms of COVID-19 will be analyzed using survival analysis. The time from randomization to complete resolution of symptoms with ivermectin vs. placebo is assessed using a Kaplan-Meier plot. Median survival time and the corresponding 95% CI will be presented. The hazard ratio and 95% CI for the cumulative incidence of symptom resolution in both treatment groups are estimated using the Cox proportional hazards model.

Evolution of hemodynamic status from days 1 to 14 will be compared with the control groups (ivermectin vs. placebo) concerning their mean change in hemodynamic status between baseline and 14 days using a linear mixed model. Study subjects will be considered as random effects, treatment group and visit number as fixed effects. Baseline value of hemodynamic status will be included as a covariate. The estimated difference in mean change from baseline to 14 days and the corresponding 95% CI will be presented.

6.3 Safety and adverse drug events

Adverse drug events will be presented as frequency and percentage.

**7. Missing data**

When analyzing using the ITT population, the last observation carried forward (LOCF) method or model based multiple imputation (MI) will be used for both primary and secondary outcomes.
